# Tissue-specific gene expression in bovine gastrointestinal tract following nonsteroidal anti-inflammatory drug–induced barrier dysfunction

**DOI:** 10.3168/jdsc.2025-0996

**Published:** 2026-03-06

**Authors:** Avery Beimer, Vinicius Gouvea, Ryan Foster, Tanner Scull, Oscar Benitez

**Affiliations:** 1 Department of Veterinary Sciences, Davis College of Agricultural Sciences and Natural Resources, Texas Tech University, Lubbock, TX 79409; 2 Texas A&M AgriLife Research, Amarillo, TX 79106; 3 School of Veterinary Medicine, Texas Tech University, Amarillo, TX 79106

## Abstract

Gastrointestinal (GI) barrier integrity is essential for nutrient absorption, health, and production efficiency in dairy cattle. Nonsteroidal anti-inflammatory drugs (NSAID) reduce prostaglandin synthesis and may compromise mucosal defenses, increasing intestinal permeability. This exploratory study characterized tissue-specific transcriptional responses to NSAID-induced barrier dysfunction in dairy heifers. Eight 13-mo-old heifers were assigned to control (n = 4) or aspirin-treated (n = 4) groups. Aspirin was administered at 100 mg/kg BW every 12 h for 4 d (d 4–7). On d 13, animals were euthanized and epithelial tissues from the rumen, small intestine, colon, and liver were collected at slaughter for RNA sequencing. A total of 370 differentially expressed genes were detected, with 355 localized to the colon, indicating a concentrated response in this tissue. Enriched pathways included cell adhesion, ion transport, extracellular matrix-receptor interaction, and PI3K-Akt signaling, suggesting activation of epithelial repair and extracellular matrix remodeling processes. These observations, though limited by sample size, highlight transcriptional changes in colon tissue following NSAID administration and provide preliminary insight into molecular pathways potentially involved in barrier dysfunction in dairy cattle.

## Graphical Abstract

**Figure:**
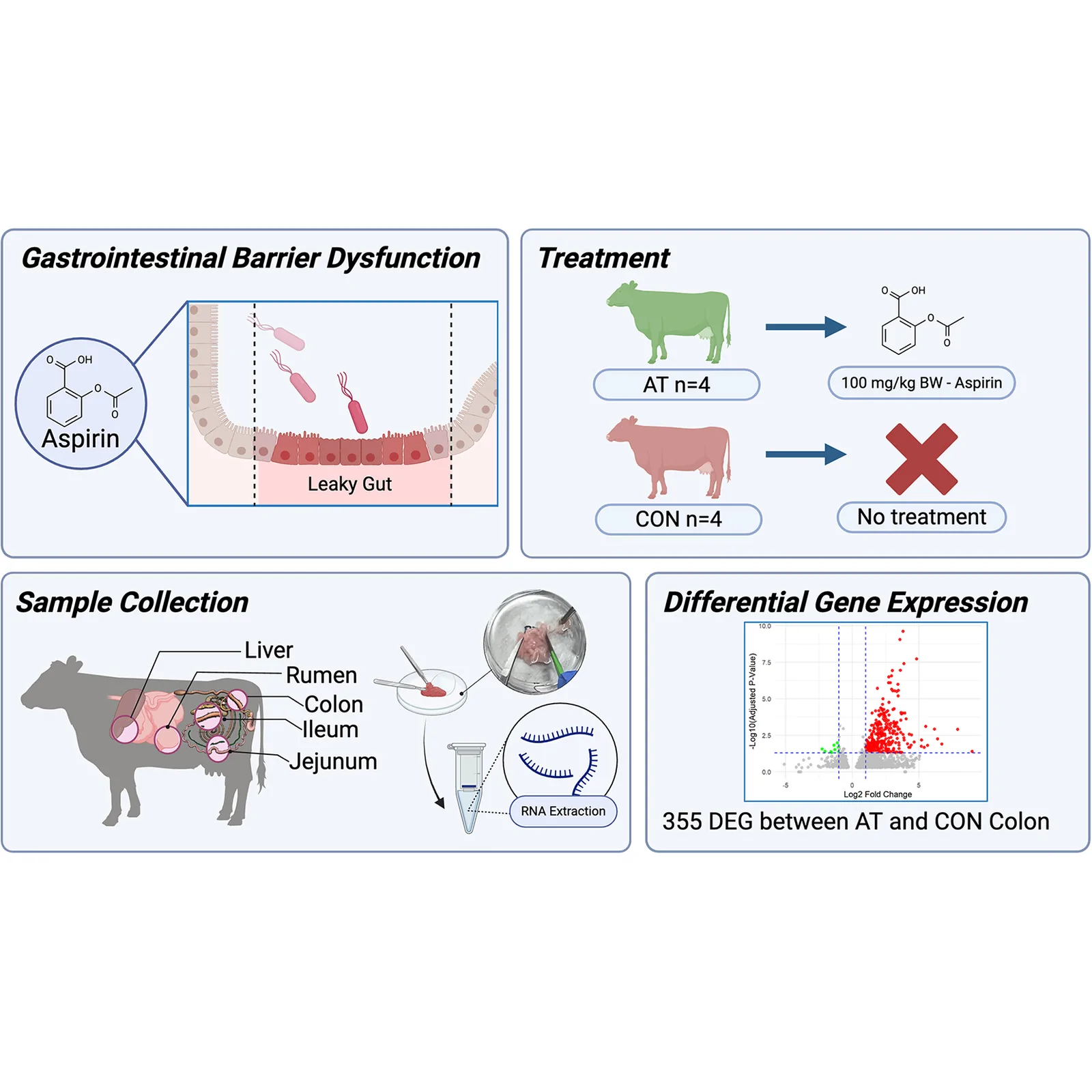

In dairy cattle, maintaining gastrointestinal (**GI**) barrier integrity is essential for nutrient absorption, health, and overall production efficiency. The protective, selectively permeable nature of the GI tract minimizes interaction between luminal microbiota and host tissues while facilitating transport of water and nutrients across the intestinal lining (Steele et al., 2016). Increased intestinal permeability, commonly referred to as “leaky gut syndrome,” is a silent, yet significant source of inefficiency in cattle production, often going unnoticed until it manifests as broader health issues (Kvidera et al., 2017). This condition has a wide scope of implications for animal welfare and productivity, as compromised barrier integrity can lead to reduced feed efficiency, slower growth rates, and a higher risk of disease (Kvidera et al., 2017). Various factors, including dietary inconsistencies (Zhang et al., 2013), ruminal acidosis (Minuti et al., 2014), and heat stress (Ghulam Mohyuddin et al., 2022), can influence the onset of leaky gut syndrome in cattle. Nonsteroidal anti-inflammatory drug (**NSAID**) treatment, commonly used for pain and inflammation management, is among the numerous factors that can exacerbate the condition (Briggs et al., 2020).

Nonsteroidal anti-inflammatory drugs, as cyclooxygenase (**COX**) inhibitors, directly reduce prostaglandin production, which plays a crucial role in the intestinal barrier function by stimulating mucus secretion, promoting blood flow, and supporting epithelial integrity (Dey et al., 2006). Reduced prostaglandin synthesis can compromise the intestinal barrier and mucosal defenses, allowing luminal bacteria and toxins to translocate paracellularly and potentially contributing to systemic inflammation and impaired metabolic function (Bjarnason and Takeuchi, 2009; Kvidera et al., 2017; Takeuchi and Amagase, 2018).

Despite the relevance of NSAID-associated barrier dysfunction to cattle health and productivity, limited research has examined the tissue-specific transcriptional responses within the bovine gastrointestinal tract, leaving the molecular pathways underlying leaky gut syndrome largely unexplored. The objective of this study was to characterize the transcriptional changes across multiple bovine gastrointestinal tissues following an NSAID challenge, using an established model of aspirin-induced barrier dysfunction (Briggs et al., 2020).

Using RNA sequencing, we compared gene expression profiles in the rumen, jejunum, ileum, colon, and liver to identify pathways disrupted by increased intestinal permeability. This approach provides an understanding of how NSAID-induced damage alters gastrointestinal epithelial defense, extracellular matrix remodeling, and host-microbe interactions, offering new insights into the molecular basis of leaky gut in cattle and potential targets for therapeutic intervention.

This transcriptomic analysis represents a secondary use of samples collected as part of an in vivo study conducted from April to October 2023 (Foster et al., 2026). The original study enrolled 15 heifers and was designed to evaluate the effects of induced gastrointestinal barrier dysfunction on systemic immune responses during a respiratory challenge. The present study was not involved in the experimental design or animal procedures of the parent study. Gastrointestinal tissue samples from a subset of animals (n = 8) that did not undergo the respiratory challenge were used for RNA sequencing and downstream analysis.

The in vivo study consisted of 13-mo-old heifers obtained from a single dairy farm. Heifers were transported to the research facility and allowed a 5-d acclimation period before the start of the experiment. Paired-day unshrunk BW was recorded at the start of the experiment (d −1 and d 1; initial BW = 475 ± 12 kg), after which heifers were blocked by BW and randomly assigned to control (n = 4), receiving no NSAID treatment, or aspirin-treated (n = 4) receiving aspirin to induce barrier dysfunction. The study was conducted in October, and all animals were individually housed under controlled environmental conditions in 1.0 × 2.5 m pens equipped with rubber flooring and automatic water fountains. Heifers were fed a basal diet consisting of a commercially available feed (Sweet Bran, Cargill, Dalhart, TX) formulated to meet the requirements of growing cattle (NASEM, 2016). Feed intake was monitored daily, and no off-feed events were observed.

Aspirin (i.e., acetylsalicylic acid; AHC Products Inc., Winchester, KY) was administered orally in 24-mL porcine hard gelatin capsules (Torpac Inc., Fairfield, NJ) via balling gun at 100 mg/kg BW every 12 h for 4 d (d 4–7; Briggs et al., 2020; Figure 1a). All animal procedures were approved by the Institutional Animal Care and Use Committee at West Texas A&M University (protocol number 2023.01.003).

**Figure 1:**
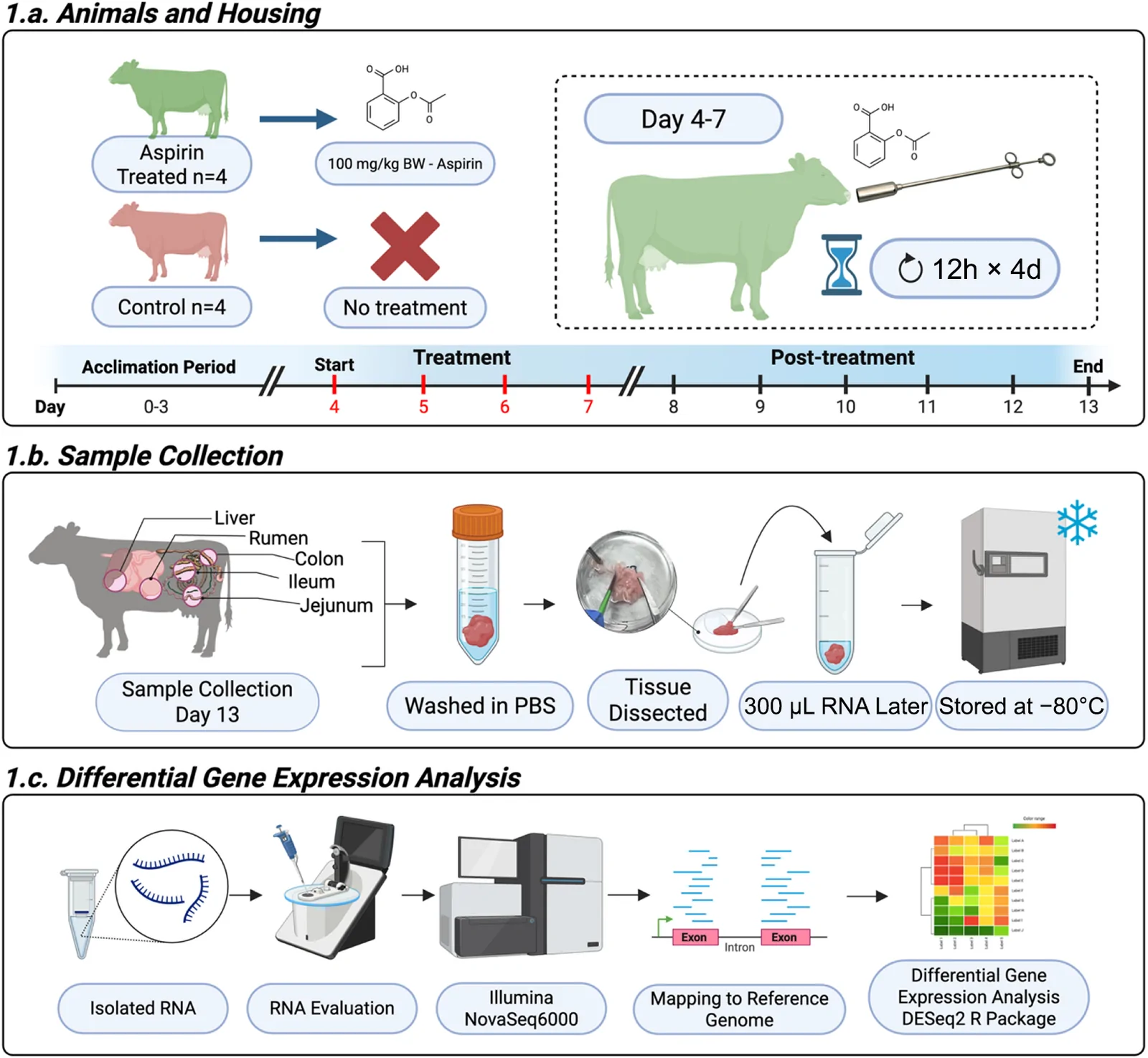
Overview of the experimental design, tissue collection, and differential gene expression analysis. (a) Eight 13-mo-old Angus × Holstein heifers were assigned to an aspirin-treated group (n = 4) or untreated control group (n = 4). Aspirin was administered orally at 100 mg/kg BW every 12 h for 4 d (d 4–7). (b) On d 13, rumen, jejunum, ileum, colon, and liver samples were collected. Intestinal tissues were washed with PBS, and epithelial-enriched tissue was manually dissected, placed in 300 µL of RNAlater Stabilization Solution, and stored at −80°C. (c) RNA was isolated using the RNeasy Mini Kit, evaluated for concentration and purity, sequenced on the Illumina NovaSeq 6000 platform, mapped to the bovine reference genome, and analyzed for differential gene expression using the DESeq2 R package. Created in BioRender. Benitez, O. (2026) https://BioRender.com/wcdz0md.

On d 13, all heifers were humanely euthanized, and intestinal tissue samples were collected from the rumen, jejunum, ileum, colon, and liver immediately following slaughter. Intestinal regions were identified using established anatomical landmarks to ensure consistency across animals. The jejunum was identified as the mid-small intestine segment distal to the duodenum, whereas the ileum was identified as the distal portion of the small intestine proximal to the ileocecal junction. Sampling locations within each region were standardized across animals.

Following collection, tissue samples were gently rinsed with PBS to remove luminal contents. To enrich for epithelial-associated transcriptional signals, the luminal epithelial layer was manually dissected from the underlying submucosa and muscularis tissue using sterile tweezers and scissors. This approach was used to minimize the inclusion of connective and muscular tissue while focusing on the mucosal epithelium. The epithelial-enriched tissue was collected and placed in 2.0-mL Eppendorf Safe-Lock Tubes containing 300 µL of RNAlater Stabilization Solution (Thermo Fisher Scientific). Samples were flash-frozen in liquid nitrogen and stored at −80°C until further processing (Figure 1b). Although complete separation of intestinal layers is not feasible using manual dissection, this method provided epithelial-enriched samples suitable for assessing transcriptional responses associated with barrier integrity.

For RNA extraction, tissue samples were thawed, and ∼30 mg of tissue was homogenized for one minute. Total RNA was then extracted from each sample using the RNeasy Mini Kit (Qiagen, Germantown, MD) following manufacturer's instructions. The RNA samples were resuspended in RNase-free water and evaluated for concentration and purity using a NanoDrop One Microvolume UV-Vis Spectrophotometer (Thermo Scientific). All RNA samples were stored at −80°C until downstream analysis. Transcriptomic analysis focused on determining tissue-specific responses to NSAID treatment relative to controls.

Messenger RNA purification was carried out using poly-T oligo-attached magnetic beads, then fragmented. First-strand cDNA was synthesized using random hexamer primers, followed by second-strand synthesis. Libraries were constructed through end repair, A-tailing, adapter ligation, and PCR amplification. Library quality was assessed using Qubit (Thermo Fisher Scientific), quantitative PCR, and Bioanalyzer (Agilent Technologies) systems. Libraries were sequenced on the Illumina NovaSeq 6000 platform (Illumina, San Diego, CA). Library preparation and sequencing were conducted by Novogene Corporation Inc. (Sacramento, CA).

Raw sequencing data were processed using fastp (https://github.com/OpenGene/fastp; Chen et al., 2018) to remove adapters and low-quality reads. Additional filtering was applied and reads with more than 10% undetermined bases (N) and reads in which over 50% of bases had Q-scores ≤ 5 were removed. Sequencing quality was further evaluated using Q20/Q30 scores, guanine-cytosine content, and base error rate metrics. Clean reads were mapped to the *Bos taurus* reference genome (Ensembl release 109; ARS-UCD1.2) using Hisat2 (v2.0.5; Center for Computational Biology at Johns Hopkins University at https://daehwankimlab.github.io/hisat2/; Kim et al., 2015). Read counts for each gene were obtained using featureCounts (v1.5.0-p3, Novogene; Liao et al., 2014), and gene expression levels were normalized as fragments per kilobase of transcript per million mapped reads (**FPKM**) to correct for differences in gene length and sequencing depth. Raw read counts were used for input to DESeq2 (Novogene; Love et al., 2014), and FPKM values were calculated for descriptive analyses and visualization, including heatmaps and principal component analysis plots to assess sample clustering.

Differential gene expression between control and aspirin-treated groups was analyzed using the DESeq2R package (1.20.0), with 4 biological replicates per group (Figure 1c). Genes with an adjusted *P*-value ≤0.05 and log2 fold change >1 were considered significantly differentially expressed. The *P*-values were adjusted by using the Benjamini–Hochberg procedure for controlling false discovery rate. Significantly differentially expressed genes were compiled into a gene set for clustering and visualization analyses. Differential expression results were visualized using volcano plots to illustrate fold change and statistical significance. Functional enrichment analysis of differentially expressed genes was performed using the clusterProfiler R package (Yu et al., 2012). Gene Ontology (**GO**) and Kyoto Encyclopedia of Genes and Genomes (**KEGG**) pathway enrichment were assessed. For GO and KEGG analyses, enrichment values were corrected within the clusterProfiler R package to account for gene length bias. Pathways or terms with adjusted *P*-values less than 0.05 were considered significantly enriched. Enrichment results were visualized using bar and dot plots to display significantly enriched GO and KEGG terms.

A total of 370 differentially expressed genes (**DEG**) were identified between aspirin-treated and control heifers (n = 4 per group) based on an adjusted *P*-value ≤0.05 and log2 fold change >1.

The colon accounted for the majority of transcriptional differences, with 355 DEG detected in the aspirin-treated group relative to control (335 upregulated and 20 downregulated). A heatmap was generated to visualize expression patterns of DEG between aspirin-treated and control colon tissue (Figure 2.a). Additionally, a volcano plot was generated to visualize the distribution of DEG in colon tissue (Figure 2.b) demonstrating a predominance of upregulated DEG. The GO enrichment analysis (clusterProfiler) of colon DEG revealed notable overrepresentation of pathways involved in cell adhesion, ion transport, and calcium ion binding. The KEGG pathway enrichment analysis further identified significantly enriched pathways such as extracellular matrix (**EXM**)-receptor interaction, PI3K-Akt signaling, calcium signaling, and regulation of actin cytoskeleton.

**Figure 2:**
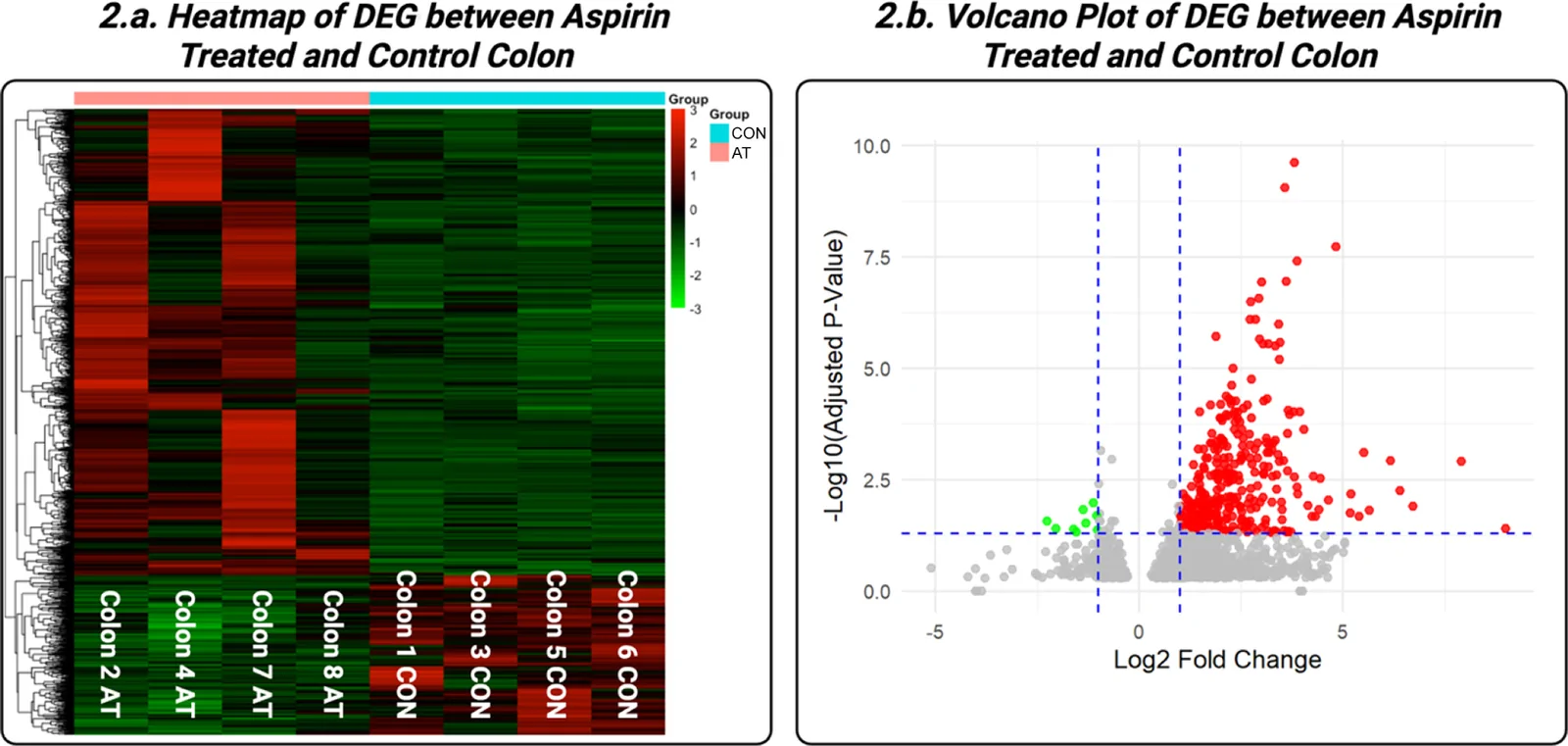
Heatmap of DEG between aspirin-treated (AT) and control (CON) colon (2.a.). Volcano plot of DEG between AT and CON colon (2.b.). Gray points represent genes that were not differentially expressed, green points represent downregulated genes, and red points represent upregulated genes.

Outside the colon, transcriptional responses were minimal, with only a small number of DEG observed in other gastrointestinal tissues. This pattern indicates that the NSAID-induced transcriptional responses were largely confined to the colon, suggesting tissue-specific sensitivity to NSAID-induced barrier disruption. In the rumen of aspirin-treated heifers, a single gene was upregulated with no downregulated genes detected. The jejunum showed 4 upregulated genes and 2 downregulated genes, while the ileum had 2 upregulated genes and no downregulated genes. The liver displayed 3 upregulated and 3 downregulated genes in aspirin-treated heifers relative to control.

Among the most significantly upregulated genes in the colon were *FLNC*, *SYNM*, *MYH11*, *SORBS1*, *ATP2B4*, and *COL6A3*, which are associated with cytoskeletal structure, contractile function, and epithelial tension. Genes involved in fibrotic remodeling and EXM reorganization, including *TNC*, *COL11A1*, *SEMA3C*, were also significantly upregulated.

Five genes, *HTR1B*, *PI15*, *CDKL5*, *ABCC4/MRP4* (*ENSBTAG00000047181*), and an uncharacterized protein coding locus (*ENSBTAG00000036061*), were exclusively expressed in the aspirin-treated samples, with no detectable expression in any control samples, suggesting treatment-specific transcriptional activation. Database searches confirmed *ABCC4* as the bovine ortholog of the multidrug resistance-associated transporter MRP4. *ENSBTAG00000036061* remains uncharacterized and lacks functional annotation or known orthologs.

Minimal differential expression was detected in the rumen, jejunum, ileum, and liver, indicating that transcriptional changes were largely restricted to the colon. The only notable exception was *B4GALNT2*, which was consistently upregulated in both the colon and small intestine. *B4GALNT2* encodes a glycosyltransferase involved in mucin glycosylation and expression of the Sda antigen (Lo Presti et al., 2003). In this dataset, the NSAID challenge elicited a predominantly colon-specific response marked by strong activation of cytoskeletal organization and extracellular matrix remodeling pathways.

This study demonstrates the colon's distinct transcriptional response to NSAID exposure when compared with the rumen, jejunum, ileum, and liver. This vulnerability has important implications for cattle receiving NSAID during illness or management procedures, an important consideration for maintaining gastrointestinal health and productivity in dairy heifers. Unlike the small intestine, which prioritizes rapid nutrient absorption, the colon is anatomically specialized for prolonged luminal retention, water absorption, and microbial fermentation. As a result, the colonic epithelium is exposed to a dense and metabolically active microbial population for extended periods, placing greater functional demands on mucosal barrier integrity, mucus production, and immune surveillance (Johansson et al., 2011). This suggests that the colon may be particularly sensitive to barrier dysfunction because its epithelial integrity depends heavily on mucosal defense and mucus barrier maintenance (Johansson et al., 2011; Pelaseyed et al., 2014), which may increase vulnerability during NSAID treatment. Because NSAID inhibit COX enzymes and reduce prostaglandin synthesis, disruption of prostaglandin-mediated mucosal defense may further increase susceptibility to epithelial injury of the colon.

The enrichment of GO terms related to cell adhesion and cytoskeletal remodeling (Table 1) supports a compensatory response aimed at restoring tight junctions and reinforcing epithelial structure following periods of extended NSAID exposure (Blikslager et al., 2007). Enrichment of ion transport and channel activity suggests that the colon is actively re-establishing ion gradients that may have been disrupted during the NSAID treatment (Deng et al., 2021). Additionally, the enrichment of extracellular matrix-related GO terms, including those linked to collagen deposition, matrix remodeling, and protease inhibition, further points to ongoing EXM turnover (Blikslager et al., 2007). Although EXM remodeling may reflect tissue repair, prolonged activation of these pathways may indicate early transcriptional signatures associated with fibrotic remodeling or reduced absorptive efficiency, both of which are concerns for gut health and nutrient utilization in dairy cattle. Taken together, the enrichment profiles suggest a coordinated biological response rather than isolated pathway activation, consistent with epithelial cells initiating coordinated repair processes to restore barrier integrity and functional homeostasis following NSAID-induced damage. The KEGG pathway analysis further supports this, highlighting upregulation of EXM-receptor interaction and PI3K-Akt signaling, which have been associated with tissue repair and fibrosis (Wang et al., 2025). Collectively, these pathway activations suggest that the colon initiates a multilayered response involving physical barrier restoration, ion transport regulation, and extracellular remodeling in an effort to recover from NSAID-induced epithelial injury.

**Table 1:** Differentially expressed genes, enriched Gene Ontology (GO) terms, and KEGG pathways identified in colon tissue of aspirin-treated and control dairy heifers^1^

Tissue	Gene ID	Ensembl ID	Padj.	Log2FC
Colon	*FLNC*	*ENSBTAG00000006253*	2.34 × 10^−10^	3.81
Colon	*SYNM*	*ENSBTAG00000011752*	8.66 × 10^−10^	3.58
Colon	*MYH11*	*ENSBTAG00000015988*	2.68 × 10^−7^	2.94
Colon	*SORBS1*	*ENSBTAG00000002880*	3.15 × 10^−7^	2.74
Colon	*ATP2B4*	*ENSBTAG00000014059*	7.81 × 10^−7^	2.86
Colon	*COL6A3*	*ENSBTAG00000030190*	3.08 × 10^−3^	1.43
Colon	*TNC*	*ENSBTAG00000000575*	1.17 × 10^−4^	2.17
Colon	*COL11A1*	*ENSBTAG00000021217*	4.78 × 10^−2^	3.63
Colon	*SEMA3C*	*ENSBTAG00000006138*	2.97 × 10^−4^	2.71
Colon	*HTR1B*	*ENSBTAG00000037429*	1.52 × 10^−2^	5.65
Colon	*PI15*	*ENSBTAG00000019587*	1.17 × 10^−3^	6.16
Colon	*CDKL5*	*ENSBTAG00000007428*	2.09 × 10^−2^	5.40
Colon	*ABCC4/MRP4*	*ENSBTAG00000047181*	4.01 × 10^−2^	8.99
Colon	*ENSBTAG00000036061*	*ENSBTAG00000036061*	1.22 × 10^−3^	7.91
JIC	*B4GALNT2*	*ENSBTAG00000009864*	1.23 × 10^−2^	6.71

	GO Term	GO ID		Count
Colon	Cell adhesion	GO:0007155	3.6 × 10^−6^	28 genes
Colon	Ion transport	GO:0006811	2.2 × 10^−3^	44 genes
Colon	Calcium ion binding	GO:0005509	8.8 × 10^−6^	50 genes

	KEGG Term	KEGG ID		
Colon	EXM-receptor interaction	bta04512	4.1 × 10^−12^	30 genes
Colon	PI3K-Atk signaling	bta04151	1.0 × 10^−3^	46 genes
Colon	Calcium signaling	bta04020	7.7 × 10^−6^	44 genes
Colon	Regulation of actin cytoskeleton	bta04810	2.7 × 10^−2^	27 genes

1 JIC = jejunum, ileum, and colon; Padj = adjusted *P*-value; Log2FC = log2 fold change.

Upregulation in genes associated with cytoskeletal structure, contractile function, and epithelial tension, including *FLNC*, *SYNM*, *MYH11*, *SORBS1*, *ATP2B4*, and *COL6A3* indicates a coordinated structural remodeling program in the aspirin-treated colon, consistent with enhanced contractile and mechanical stress responses. Additionally, upregulation of *TNC*, *COL11A1*, and *SEMA3C*, which are involved in fibrotic remodeling and EXM reorganization (Midwood et al., 2016), supports the interpretation that epithelial barrier disruption is accompanied by extracellular matrix remodeling processes that may contribute to tissue repair and early fibrotic signaling.

Five genes were uniquely expressed in the aspirin-treated group, namely *HTR1B*, *PI15*, *CDKL5*, *ABCC4/MRP4* (*ENSBTAG00000047181*), and *ENSBTAG00000036061*, with no detectable expression in any control samples. Collectively, these genes represent potential molecular markers of NSAID-induced mucosal stress, offering targets for studying drug safety and gut recovery mechanisms in cattle. *HTR1B*, which encodes a G-protein coupled receptor for serotonin (5-hydroxytryptamine or 5-HT), was upregulated in the aspirin-treated colon. Serotonin release activates the enteric nervous system (**ENS**) to increase motility and modulate inflammation, which may explain its upregulation in the aspirin-treated group. However, it remains unclear whether serotonergic activation supports mucosal repair or contributes to dysregulated motility and inflammation. Nonsteroidal anti-inflammatory drug–induced barrier dysfunction can promote mucosal stress and immune activation, both of which are known triggers for altered serotonergic signaling (Kendig and Grider, 2015). Increased *HTR1B* expression may represent a compensatory mechanism to regulate motility and inflammation under conditions of colonic injury or alternatively reflect dysregulated ENS signaling as part of the injury response (Gershon, 2013). *PI15* (Peptidase inhibitor 15), a trypsin-like protease inhibitor, may reflect a protective response aimed at limiting protease-mediated damage to the EXM and tight junctions (Biancheri et al., 2013). Given the colon's dense microbial load and prostaglandin-mediated defense, *PI15* may represent a mediator of mucosal stress response. Additionally, *CDKL5* (cyclin-dependent kinase-like 5), was uniquely upregulated in the aspirin-treated colon and was not detected in control samples. *CDKL5*, primarily characterized in the central nervous system of humans (Fehr et al., 2013), has not been well characterized in gastrointestinal tissue. Its unique upregulation in aspirin-treated colon suggests a novel role in epithelial stress or repair signaling. *ABCC4/MRP4* (*ENSBTAG00000047181*), the bovine ortholog of the multidrug resistance transporter, mediates prostaglandin transport (Fonseca-Camarillo et al., 2025) and may represent a critical component of mucosal defense. In contrast, *ENSBTAG00000036061* remains uncharacterized, underscoring the presence of potentially novel stress-responsive loci in bovine colon.

Although the colon exhibited the strongest response, transcriptional changes in other tissues were limited, but may still reflect subtle systemic adaptations. *B4GALNT2* was consistently upregulated in both colon and small intestine tissues. This gene, associated with glycosylation and the Sda antigen, may influence mucin composition and host-microbe interactions (Lo Presti et al., 2003), suggesting a shared glycosylation-related response to NSAID-induced barrier dysfunction across intestinal regions.

This study provides exploratory transcriptomic insight into tissue-specific responses to NSAID exposure in cattle. Collectively, these findings highlight the colon as the primary site of NSAID-induced transcriptional disruption, characterized by cytoskeletal remodeling, EXM reorganization, and activation of epithelial defense pathways. The identification of treatment-specific genes, including *PI15*, *CDKL5*, *HTR1B*, and *ABCC4/MRP4*, provides new insights into molecular mechanisms of gastrointestinal barrier dysfunction and suggests potential targets for mitigating NSAID-induced intestinal injury. Future studies should determine whether these transcriptional responses translate into measurable physiological outcomes and explore how management or nutritional interventions might reduce NSAID-associated gut injury in dairy cattle. These findings provide a molecular foundation for improving gastrointestinal health and productivity in livestock receiving NSAID treatment.

Because aspirin has a short half-life and gastrointestinal epithelial turnover is rapid, tissue collection 6 d after treatment withdrawal likely reflects downstream transcriptional responses associated with barrier disruption and repair rather than acute pharmacologic effects. Earlier molecular responses may therefore have been missed, representing a limitation of the study. An additional limitation of this study relates to the control procedures used for capsule administration. Control animals did not receive empty gelatin capsules. This approach was intended to minimize handling and balling gun–associated stress. Because stress itself is known to influence gastrointestinal barrier integrity, the absence of a control capsule should be considered when interpreting NSAID-specific transcriptional responses.

## References

[bib1] Biancheri P., Di Sabatino A., Corazza G.R., MacDonald T.T. (2013). Proteases and the gut barrier. Cell Tissue Res..

[bib2] Bjarnason I., Takeuchi K. (2009). Intestinal permeability in the pathogenesis of NSAID-induced enteropathy. J. Gastroenterol..

[bib3] Blikslager A.T., Moeser A.J., Gookin J.L., Jones S.L., Odle J. (2007). Restoration of barrier function in injured intestinal mucosa. Physiol. Rev..

[bib4] Briggs N.G., Brennan K.M., Funnell B.J., Nicholls G.T., Schoonmaker J.P. (2020). Use of aspirin to intentionally induce gastrointestinal tract barrier dysfunction in feedlot cattle. J. Anim. Sci..

[bib5] Chen S., Zhou Y., Chen Y., Gu J. (2018). fastp: An ultra-fast all-in-one FASTQ preprocessor. Bioinformatics.

[bib6] Deng Z., Zhao Y., Ma Z., Zhang M., Wang H., Yi Z., Tuo B., Li T., Liu X. (2021). Pathophysiological role of ion channels and transporters in gastrointestinal mucosal diseases. Cell. Mol. Life Sci..

[bib7] Dey I., Lejeune M., Chadee K. (2006). Prostaglandin E2 receptor distribution and function in the gastrointestinal tract. Br. J. Pharmacol..

[bib8] Fehr S., Wilson M., Downs J., Williams S., Murgia A., Sartori S., Vecchi M., Ho G., Polli R., Psoni S., Bao X., de Klerk N., Leonard H., Christodoulou J. (2013). The CDKL5 disorder is an independent clinical entity associated with early-onset encephalopathy. Eur. J. Hum. Genet..

[bib9] Fonseca-Camarillo G., Furuzawa-Carballeda J., Miguel-Cruz E., Barreto-Zuñiga R., Martínez-Benítez B., Yamamoto-Furusho J.K. (2025). Protective role of ABCC drug subfamily resistance transporters (ABCC1-7) in intestinal inflammation. Immunol. Res..

[bib10] Foster R.C., Gouvêa V.N., Beck M.R., Benitez O.J., Diaz-Delgado J., Migl K.F., Rosa F., Scott M.A., Long N.S., Pennington J.T., Cooke R.F. (2026). Effects of gut barrier dysfunction during a viral respiratory disease challenge on immune function of feedlot beef calves. J. Anim. Sci..

[bib11] Gershon M.D. (2013). 5-Hydroxytryptamine (serotonin) in the gastrointestinal tract. Curr. Opin. Endocrinol. Diabetes Obes..

[bib12] Ghulam Mohyuddin S., Khan I., Zada A., Qamar A., Arbab A.A.I., Ma X.-B., Yu Z.-C., Liu X.-X., Yong Y.-H., Ju X.H., Zhang-Ping Y., Yong Jiang M. (2022). Influence of heat stress on intestinal epithelial barrier function, tight junction protein, and immune and reproductive physiology. BioMed Res. Int..

[bib13] Johansson M.E., Larsson J.M.H., Hansson G.C. (2011). The two mucus layers of colon are organized by the MUC2 mucin, whereas the outer layer is a legislator of host–microbial interactions. Proc. Natl. Acad. Sci. USA.

[bib14] Kendig D.M., Grider J.R. (2015). Serotonin and colonic motility. Neurogastroenterol. Motil..

[bib15] Kim D., Langmead B., Salzberg S. (2015). HISAT: A fast spliced aligner with low memory requirements. Nat. Methods.

[bib16] Kvidera S.K., Dickson M.J., Abuajamieh M., Snider D.B., Sanz Fernandez M.V., Johnson J.S., Keating A.F., Gorden P.J., Green H.B., Schoenberg K.M., Baumgard L.H. (2017). Intentionally induced intestinal barrier dysfunction causes inflammation, affects metabolism, and reduces productivity in lactating Holstein cows. J. Dairy Sci..

[bib17] Liao Y., Smyth G.K., Shi W. (2014). featureCounts: An efficient general-purpose program for assigning sequence reads to genomic features. Bioinformatics.

[bib18] Lo Presti L., Cabuy E., Chiricolo M., Dall'Olio F. (2003). Molecular cloning of the human β1,4 N-acetylgalactosaminyltransferase responsible for the biosynthesis of the Sd^a^ histo-blood group antigen: The sequence predicts a very long cytoplasmic domain. J. Biochem..

[bib19] Love M.I., Huber W., Anders S. (2014). Moderated estimation of fold change and dispersion for RNA-seq data with DESeq2. Genome Biol..

[bib20] Midwood K.S., Chiquet M., Tucker R.P., Orend G. (2016). Tenascin-C at a glance. J. Cell Sci..

[bib21] Minuti A., Ahmed S., Trevisi E., Piccioli-Cappelli F., Bertoni G., Jahan N., Bani P. (2014). Experimental acute rumen acidosis in sheep: Consequences on clinical, rumen, and gastrointestinal permeability conditions and blood chemistry. J. Anim. Sci..

[bib22] NASEM. (National Academies of Sciences, Engineering, and Medicine) (2016).

[bib23] Pelaseyed T., Bergström J.H., Gustafsson J.K., Ermund A., Birchenough G.M., Schütte A., van der Post S., Svensson F., Rodríguez-Piñeiro A.M., Nyström E.E., Wising C., Johansson M.E.V., Hansson G.C. (2014). The mucus and mucins of the goblet cells and enterocytes provide the first defense line of the gastrointestinal tract and interact with the immune system. Immunol. Rev..

[bib24] Steele M.A., Penner G.B., Chaucheyras-Durand F., Guan L.L. (2016). Development and physiology of the rumen and the lower gut: Targets for improving gut health. J. Dairy Sci..

[bib25] Takeuchi K., Amagase K. (2018). Roles of cyclooxygenase, prostaglandin E2 and EP receptors in mucosal protection and ulcer healing in the gastrointestinal tract. Curr. Pharm. Des..

[bib26] Wang S., Wang L., Lin J., Wang M., Li J., Guo Q., Jiao C., Tang N., Ma J., Zhang H., Zhao X. (2025). Inflammatory monocyte-derived amphiregulin mediates intestinal fibrosis in Crohn's disease by activating PI3K/AKT. Mucosal Immunol..

[bib27] Yu G., Wang L.-G., Han Y., He Q.-Y. (2012). clusterProfiler: An R package for comparing biological themes among gene clusters. OMICS J. Integrative Biol..

[bib28] Zhang S., Albornoz R., Aschenbach J., Barreda D., Penner G. (2013). Short-term feed restriction impairs the absorptive function of the reticulo-rumen and total tract barrier function in beef cattle. J. Anim. Sci..

